# Supplementary material for: A Global Screen for Assembly State Changes of the Mitotic Proteome by SEC-SWATH-MS
Source: Cell Syst. 2020 Feb 26;10(2):133–155.e6. doi: 10.1016/j.cels.2020.01.001 (PMC7042714; doi:10.1016/j.cels.2020.01.001)

Q9NPJ3 | ACO13_HUMAN | ACOT13 THEM2 HT012 PNAS−27
Monomer MW [kDa]: 14.96 Monomer expected elution fraction: 57

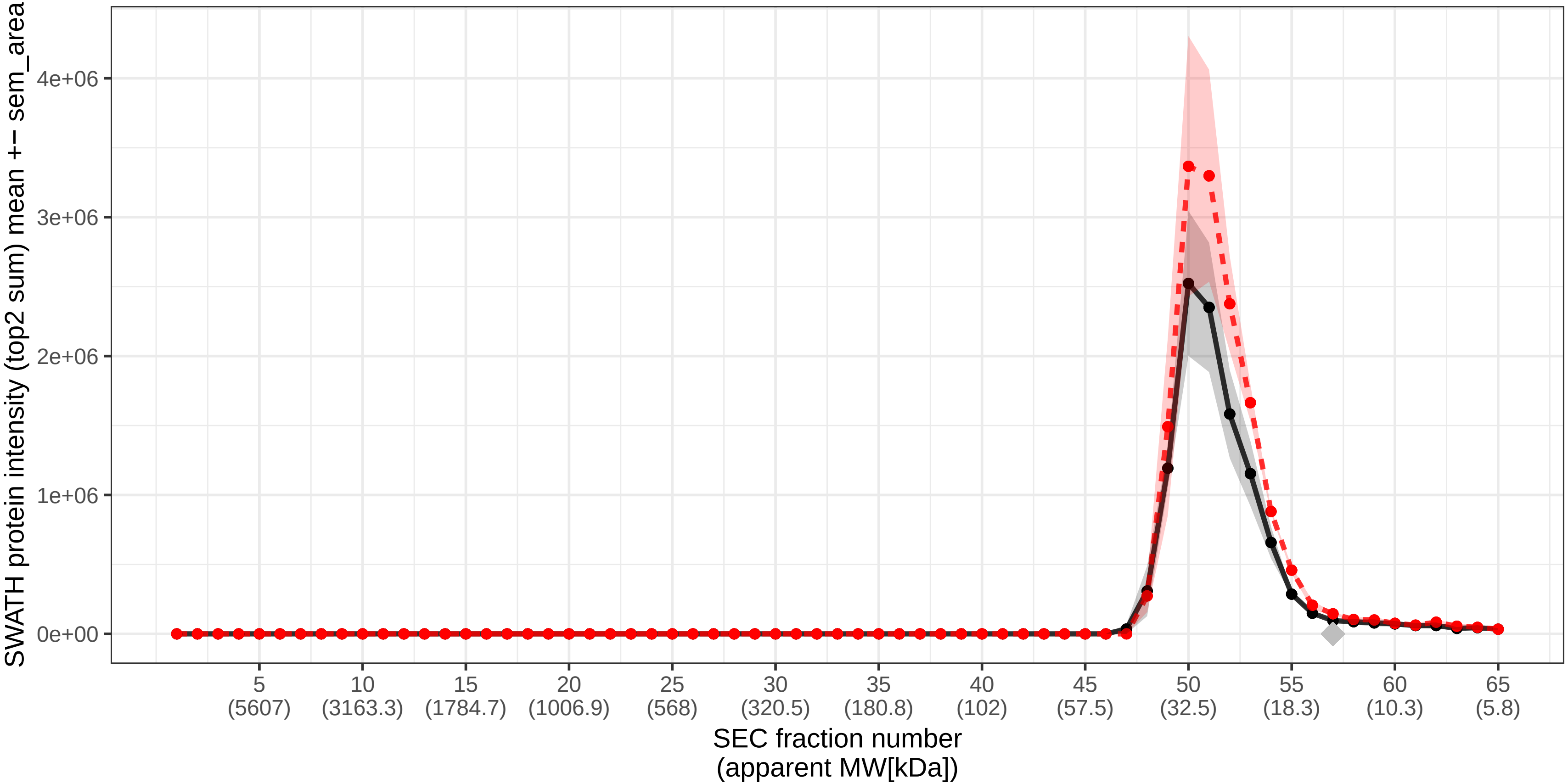

Supplement: Data S1. SEC-SWATH-MS Protein Chromatograms, Related to Figure 1 [file mmc6.zip › SECchrom_Q9NPJ3_ACO13_HUMAN_ACOT13_THEM2_HT012_PNAS-27.pdf]
